# Proportions of IgA antibodies targeting glycosylated epitopes of secreted *Escherichia coli* mucinase YghJ in initial plasmablast response differ from salivary and intestinally secreted IgA

**DOI:** 10.1007/s00430-024-00812-0

**Published:** 2024-12-14

**Authors:** Saman Riaz, Hans Steinsland, Ann Z. Andersen, Anders Boysen, Kurt Hanevik

**Affiliations:** 1 Department of Clinical Science, University of Bergen, Bergen, Norway; 2 Centre for Intervention Science in Maternal and Child Health (CISMAC), Department of Global Public Health and Primary Care, Centre for International Health, University of Bergen, Bergen, Norway; 3 Department of Biomedicine, University of Bergen, Bergen, Norway; 4 GlyProVac ApS, Rørhatten 4, Odense, Denmark; 5 National Centre for Tropical Infectious Diseases, Department of Medicine, Haukeland University Hospital, Bergen, Norway

**Keywords:** IgA, SslE, YghJ, Enterotoxigenic *Escherichia coli*, Glycosylated epitopes, TW10722

## Abstract

Mucosal infections normally cause an immune response including activation of antigen-specific B cells in regional mucosa-associated lymphoid tissue. After recirculation of plasmablasts, and maturation at mucosal surfaces or bone marrow, plasma cells produce secretory or systemic IgA. It remains uncertain to what extent secretory and systemic IgA share the same target specificities. For vaccine candidate optimization, it is important to know whether IgA targeting of glycosylated epitopes of a protein antigen vary between mucosal and systemic sites. We evaluated glycosylated epitope specificity of systemic and mucosally secreted IgA against YghJ, a potential vaccine candidate antigen secreted by most pathogenic *Escherichia coli*. IgA from intestinal lavage, saliva, serum, and blood-derived antibody in lymphocyte supernatants (ALS) were collected from 21 volunteers following experimental infection with enterotoxigenic *E. coli*. Methods for preparing IgA from saliva and ALS were developed, and multiplex bead flow cytometric immunoassays were used to determine levels of IgA targeting natively glycosylated YghJ and estimating what proportion of these antibodies specifically targeted glycosylated epitopes*.* Following infection, anti-YghJ IgA levels increased substantially for most volunteers across all four specimen types. Target specificity of ALS IgA correlated well with serum IgA, but not with mucosally secreted IgA. Furthermore, glycosylation-specific proportion of salivary IgA was higher than, and did not correlate with, intestinally secreted IgA. These results indicate a new degree of complexity to our understanding of epitope-targeting and tissue specificity of mucosal antibody responses. Our findings also suggest that all features of an intestinal IgA response may not be well reflected in serum, saliva, or ALS, which are commonly used proxy specimens for evaluating intestinal immune responses.

**Supplementary Information:**

The online version contains supplementary material available at 10.1007/s00430-024-00812-0.

## Introduction

Mucosal surfaces serve as the main portal of entry and site of colonization for most human pathogens [1, 2]. The mucosal immune system, constituting the largest component of the human immune system, protects against mucosal infections mainly through local secretion of IgA antibodies [3, 4]. Mucosally secreted IgA antibodies are mostly dimeric and produced by clones of B cells that have been activated in response to a mucosal infection or vaccination while residing in the regional mucosa associated lymphoid tissue [5–8]. These activated B cells divide prolifically, and resulting plasmablast clones recirculate to find their way to the mucosal tissues [9, 10], with some becoming long-lived memory B-cells residing in the bone marrow. The concentration of recirculating plasmablasts in blood tends to peak at around 7 days after the initial exposure [11]. Since plasmablasts found circulating in the blood are thought to be representative of plasmablasts that end up secreting IgA in the mucosal tissues, IgA isolated from the blood plasmablasts [12, 13], or so-called antibody in lymphocyte supernatants (ALS), are often used as a proxy for studying mucosal IgA responses [14, 15].

Systemic immune responses are also important for preventing and clearing mucosal infections [16, 17], and antibodies generated from a systemic immune response are mainly produced by bone marrow plasma cells [10, 18]. Although, little is known about the relationship between mucosal and systemic antibody responses, there are some indications that their antigen target-specificities overlap to a large extent [18]. For this reason, serum antibodies are also often used as a proxy for studying mucosal IgA responses [19, 20].

Enterotoxigenic *E. coli* (ETEC), which mainly cause acute and self-limiting diarrhea [13], non-invasively colonizes the small intestinal cell wall with the help of its conserved, glycosylated mucinase YghJ, which helps to break down the cell wall's mucus layer [21–23]. YghJ, also named Secreted and surface-associated lipoprotein from *E. coli* (SslE), is secreted by most pathogenic *E. coli* during colonization and is currently being targeted for vaccine development [24]. Immunization with YghJ has already been shown to protect mice against sepsis [25] and colonization [26] with pathogenic *E. coli*. In addition, both natural and experimental infections with *E. coli* tend to induce strong anti-YghJ immune responses [21, 27, 28]. Since YghJ is naturally glycosylated, there is currently a focus on whether vaccine antigens also need to be glycosylated in order to induce a protective antibody response [29], including to what extent anti-YghJ responses target glycosylated epitopes.

Results from a recent experimental human ETEC infection study suggested that the anti-YghJ IgA secreted in the gut seemed to mainly target non-glycosylated epitopes, while the corresponding serum IgA had a higher proportion targeting glycosylated epitopes. Given that IgA secreted in the gut are thought to be produced by the same antigen-specific plasmablasts that also produce systemic IgA [18], it was surprising that the intestinal and serum IgA did not have similar target specificities. Hence, using YghJ as a relevant case example, we aimed to evaluate whether mucosal anti-YghJ IgA, in general, tend to target non-glycosylated epitopes. We developed methods for preparing IgA from saliva and ALS specimens from the same 21 volunteers and compared the magnitudes and target-specificities of these to the intestinal and serum anti-YghJ IgA.

## Materials and methods

### Experimental ETEC infection study

The present study is based on data and specimens collected during an already completed human volunteer experimental infection study performed in the Division for Infectious Diseases ward at the Department of Medicine at Haukeland University Hospital, Bergen, Norway, between 2014 and 2018 [27]. In that study, 21 healthy adult volunteers aged between 19 and 28 years were experimentally infected with ETEC strain TW10722 (O115:H5; GenBank BioProject: PRJNA59745) [30], by receiving doses between 1 × 10^6^ to 1 × 10^10^ colony forming units (CFU) and followed for up to 10 days with collection of symptoms data and specimens. The infection was cleared 5 days after dose ingestion by treatment with ciprofloxacin, or earlier if a volunteer experienced severe diarrhea or moderate diarrhea lasting for ≥ 24 h. ETEC strain TW10722 encodes ETEC colonization factors Coli Surface antigen 5 (CS5) and CS6, the human variant of the heat-stable enterotoxin (STh) [30], as well as YghJ.

### ALS preparation

ALS sampling was done a few hours prior to dose ingestion (Day 0) and 7 days after, at which time the recirculating blood plasmablast concentrations are usually peaking. ALS was derived from cultures of peripheral blood mononuclear cells (PBMCs) isolated from venous blood collected in BD Vacutainer CPT Mononuclear Cell Preparation Tubes (BD Biosciences, Franklin Lakes, NJ). After centrifuging at 1800 × g for 20 min at room temp, the PBMC-containing fraction was extracted by pipetting and added to 15 mL Phosphate Buffered Saline ([PBS] 10 mM Na_2_HPO_4_, 1.8 mM KH_2_PO_4_, 2.7 mM KCl, 137 mM NaCl, pH 7.4), centrifuged at 300 × g for 15 min, and washed once with 15 mL PBS before re-centrifugation and resuspending the resulting pellet in 10 mL complete RPMI 1640 medium (Thermo Fisher Scientific, Waltham, MA).

The lymphocyte concentration in this cell suspension was determined by incubating 10 µL of the cell suspension with CD45-FITC (BD Biosciences) to stain all leukocytes, and CD14-PC5 (Beckman Coulter, Brea, CA) to stain all monocytes. After adding AccuCheck Counting Beads (BD Biosciences), the concentration of lymphocyte cells, identified CD45-FITC-positive and CD14-PC5-negative cells was determined by analysis on a C6 Accuri flow cytometer (BD Biosciences). The cell suspension was subsequently re-centrifuged and the resulting pellet was resuspended to a concentration of 1 × 10^7^ lymphocytes/mL in complete RPMI 1640 medium containing 10% heat-inactivated fetal bovine serum, 2 mM L-glutamine, and 50 µg/mL gentamicin sulfate. One mL was incubated for 72 h in a 24-well microplate without antigenic stimulation in an atmosphere of 5% CO_2_, humidified at 37°C. The resulting supernatant, representing ALS, was stored frozen at – 80 °C until analyses.

### Saliva sample collection and preparation

Saliva specimens were collected on the day of dose ingestion and 10 days after. Whole saliva was collected for the first 9 volunteers, while sublingual saliva was collected for the last 12 because a new study suggested that IgA antibodies in sublingual saliva provide a better reflection of the IgA secreted in the gut in response to an ETEC infection [14]. To collect the whole saliva, volunteers placed Salimetrics Oral Swabs (Salimetrics LLC, State College, PA) under the tongue for up to 15 min without salivary stimulation. Collection of sublingual saliva was done the same way, except that the parotid ducts were first blocked by using dental cotton rolls. The saturated swabs were then placed in Salimetrics Swab Storage Tubes (Salimetrics) and centrifuged at 3000 × g at 4 °C for 10 min. The supernatants were frozen at -80 °C after total IgA concentration determination, which was done by using the Human IgA Flex Set Kit (BD Biosciences) following the manufacturer’s protocol. The total IgA concentration estimates were later used to normalize ETEC-specific saliva anti-YghJ IgA level estimates to enable comparison of responses between volunteers and between timepoints [14, 31].

Before analyses, thawed specimens were centrifuged at 16,000 × g for 3 min, and to minimize background assay signals, any remaining mucoid aggregates were removed from the resulting supernatant by performing size-exclusion chromatography. For this procedure, Sephadex® G-25 Superfine gel filtration media (Cytiva, GmbH, Germany) was allowed to swell overnight in PBS, and 300 µL of the resulting media slurry was added to each well of a Nunc™ 278011 fritted 96-well DeepWell Filter Plates (Thermo Fisher Scientific). The plate was centrifuged at 300 × g for 1 min before washing by adding 80 µL PBS to the top of each column and re-centrifuging. Finally, 80 µL saliva supernatant was added to the top of the column, and after centrifuging the plate, the flow-through was diluted 1:8 in PBS, and immediately used in the bead-based immunological assays described below.

### Intestinal lavage and serum collection and preparation

The collection and treatment of serum and intestinal lavage specimens has been described previously [28]. Briefly, venous blood was collected on the day before and 10 days after dose ingestion, and serum was obtained by centrifugation. The intestinal lavage specimens were collected around 2 weeks prior to dose ingestion and 10 days after. For these specimens, the volunteers drank a polyethylene glycol (PEG)-based laxative until they excreted clear, watery stools. Protease inhibitors were added to the stools before they were centrifuged, sterile filtered, and stored at – 80 °C.

### YghJ production

The current study is based on the same glycosylated and non-glycosylated YghJ (gYghJ and nYghJ, respectively) preparations that were also used in a previous study [28]. In brief, to produce the glycosylated version of the YghJ, a 3xFLAG reporter gene was added to the YghJ gene in wild-type TW10722 to facilitate purification. For the non-glycosylated version, the 3xFLAG tagged YghJ gene was cloned into an expression vector and expressed in *E. coli* strain MG1655Δ*hldE*, which lacks the ability to synthesize the heptose glycans that are needed for *E. coli* protein glycosylation [32]. Both proteins were purified by using affinity chromatography and dialysis. The quality of the YghJ was tested by SDS-PAGE and quantified by using the Bicinchoninic Acid (BCA) assay [33]. The glycosylation patterns of gYghJ were characterized by using the beta-elimination of O-linked glycans followed by Michael-addition of a phosphonic acid derivative (BEMAP) analysis [28].

### Anti-YghJ IgA assay

Anti-YghJ IgA levels were estimated by using a bead-based flow-cytometry assay as previously described [28]. Purified gYghJ and nYghJ were independently covalently coupled to 4 µm Cyto-Plex (Thermo Fisher Scientific) or MagPlex carboxylated beads (Luminex Corp, Austin, TX) as described earlier [28]. Cyto-Plex beads were used to analyze the ALS specimens, while the more expensive MagPlex beads were used for saliva analyses because initial tests indicated that these beads gave lower background signals when analyzing saliva. Specimens from different timepoints belonging to the same volunteer were analyzed on the same microplate. Around 5000 each of gYghJ and nYghJ-coupled beads were used for all assays.

Prior to analyses, ALS specimens were thawed and diluted 1:5 in Assay buffer (PBS containing 1% bovine serum albumin [BSA], and 0.05% Tween-20). Wells of MultiScreen HTS HV filter plates (Merck KGaA, Darmstadt, Germany) were wetted with 50 µL Assay buffer, followed centrifugation at 300 × g for 45 sec immediately before adding 50 µL Assay buffer containing nYghJ and gYghJ-coupled beads and 50 µL diluted ALS specimen. After incubating the plates for 30 min at room temperature on a microplate shaker at 600 rpm at room temperature, the filter plates were centrifuged at 50 × g for 45 sec. Later, the beads were washed twice by adding 200 µL Assay buffer followed by re-centrifugation. After a final 30 min incubation with 50 µL Assay buffer containing 1:400 diluted Alexa Fluor 488 AffiniPure Goat Anti-Human Serum IgA antibody (Jackson ImmunoResearch, West Grove, PA), the beads were washed, as described above, twice with 200 µL Assay buffer. Beads were resuspended in 200 µL Assay buffer and analysed by flow cytometry on a LSR Fortessa flow cytometer (BD Biosciences).

As a measure of anti-YghJ IgA levels, we calculated the median fluorescence intensity (MFI) of the gYghJ-coupled beads by using FlowJo, version 10.4.2 (BD Biosciences). Each flow-cytometry analysis included a tenfold dilution series (in duplicate) of a specimen that had high anti-YghJ IgA levels, and the mean of resulting MFI standard curves generated from those dilution series were used for normalizing the estimated MFI, with the maximum set to 10,000 arbitrary units (AUs). The nYghJ-coupled beads were included in the assay merely for quality control purposes, and data from these beads were not included in any of the data analyses. To estimate the fold change in anti-YghJ antibody levels between before and after dose ingestion, we divided the estimated post-infection IgA levels with the corresponding pre-infection levels. Volunteers who had a ≥ 2.0-fold increase were considered to be responders.

Saliva specimens were analysed and responders were defined as explained for ALS, above, except that the MagPlex beads were first suspended in 20 µL PBS containing 5% BSA and 0.25% Tween-20 in a 96-well Bio-Plex Pro™ Flat Bottom Plate (Bio-Rad, Hercules, CA), followed by adding 80 µL of the diluted saliva, and that the washes were done by pipet-aspiration of liquid after immobilizing the beads on a Luminex Magnetic Plate Separator (Luminex). After calculating the AU, the estimates were further normalized by dividing with the total IgA concentration for the given specimen, and the resulting normalized AU (nAU) estimates were used in data analyses.

### Glycosylation-specific proportion assay

A glycosylation-specific proportion (GSP) assay was performed to evaluate the degree to which anti-YghJ antibody responses specifically targeted glycosylated epitopes [28]. In this competition-based assay, an excess of unbound gYghJ or nYghJ is added to the specimens prior to performing the above-described anti-YghJ IgA assay in order to selectively hinder different types of anti-YghJ IgA in the specimens to bind to the bead-coupled YghJ. Unbound gYghJ exposes both glycosylated and non-glycosylated epitopes, while nYghJ only exposes non-glycosylated epitopes [28], so treating with an excess of gYghJ in effect removes all YghJ-specific antibodies from the specimen, while treating with nYghJ only removes antibodies that bind to non-glycosylated YghJ epitopes, leaving antibodies that bind to glycosylated YghJ epitopes free to react with the bead-bound YghJ. Subtracting the IgA levels after gYghJ treatment (background level) from those after nYghJ-treatment (levels of IgA targeting glycosylated YghJ epitopes) and untreated specimens (levels of IgA targeting any YghJ epitopes) and subsequently dividing the latter two thus gives the estimated proportion of all anti-YghJ antibodies that target glycosylated epitopes (i.e. the GSP estimate). When estimating GSP for one isotype (e.g. IgA), other isotypes (e.g. IgG and IgM) may interfere with analyses and may need to be removed before analyses.

The GSP assays were performed as detailed earlier [28], except that when analyzing ALS, we added M_W_ 89,000–98,000 polyvinyl alcohol (Sigma-Aldrich, St. Louis, MO) to the Assay buffer to a 2.0% final concentration to help increase recovery of IgA when removing IgG and IgM.

### Statistical analyses

Prism, version 10, (GraphPad Software, San Diego, CA) was used for statistical analyses and for preparing graphs. Wilcoxon signed rank test were used to test for differences in anti-YghJ IgA antibody levels, GSP, and total IgA content between specimens collected from the same volunteers but at different timepoints. Mann–Whitney *U* test was used to test for differences in anti-YghJ IgA antibody levels between specimens collected from volunteers who developed diarrhea and those who did not, between volunteers who contributed whole saliva to those who contributed sublingual saliva, and in GSP differences between saliva and lavage specimens. To evaluate whether the fold change increase in anti-YghJ IgA correlated between different types of specimens, we calculated Pearson correlation coefficients. Lastly, IBM SPSS (IBM SPSS Statistics, Chicago, IL), version 29, was used to determine agreement between GSPs of different specimens using Lin’s concordance correlation coefficients.

### Ethical approval

The study (NCT02870751 at ClinicalTrials.gov) was approved by the Regional Committee for Medical and Health Research Ethics, Health Region West (REC-West; case number 2014/826). All volunteers signed written informed consent to participate in the study.

## Results

In this study, we compared levels and target specificities of anti-YghJ IgA found in specimens collected from 21 volunteers who had been experimentally infected with ETEC strain TW10722, 10 (48%) of whom developed diarrhea. New in this study are ALS and saliva estimates. We compare these to previously published estimates of intestinal lavage (19 volunteers/14 responders) and serum (21 volunteers/20 responders), where responders were defined as volunteers mounting a ≥ twofold increase in anti-YghJ IgA levels from before until 10 days after dose ingestion [28]. Two volunteers who didn't contribute lavage data were excluded because only small amounts of IgA could be detected in one or both of their intestinal lavage specimens.

### Anti-YghJ IgA levels and fold changes

**Fig. 1:**
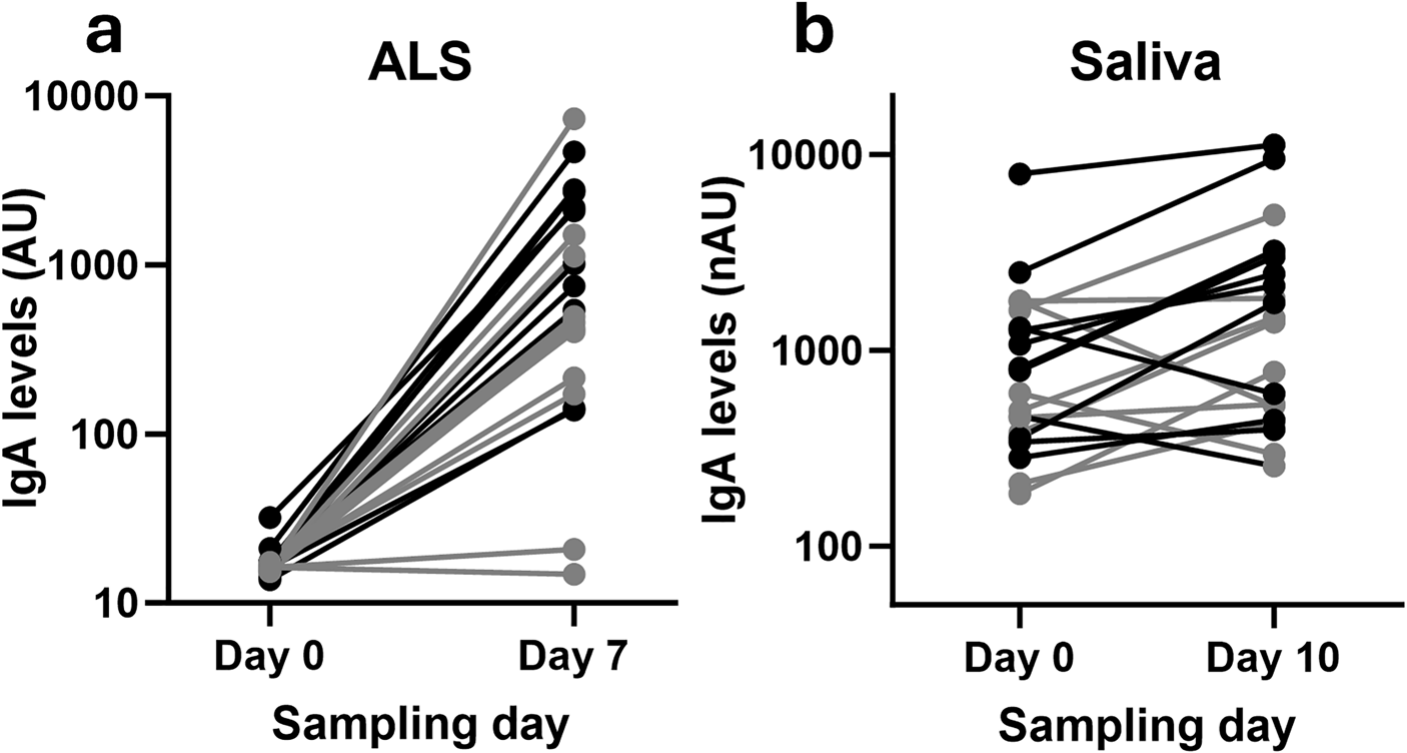
Anti-YghJ IgA antibody level changes in ALS and saliva from before and after dose ingestion. Black and grey lines represent volunteers who did and did not develop diarrhea, repectively. IgA levels are expressed as arbitrary units (AU) for ALS and normalized arbitrary units (nAU) for saliva

**Fig. 2:**
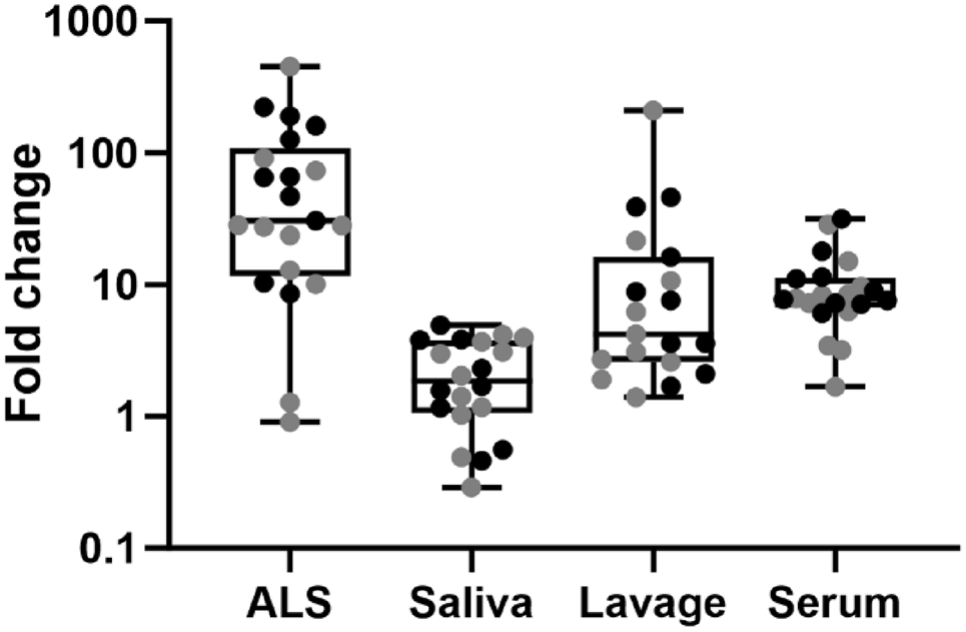
Boxplots of fold changes in anti-YghJ IgA levels for different specimen types after ETEC infection. Line in the boxes represents median values and boxes the values between 25 and 75th percentiles. The upper and lower whiskers limit 95% of measured values. Black and grey dots represent volunteers who did and did not develop diarrhea, respectively

On Day 0, the ALS anti-YghJ IgA levels were low and varied little between volunteers (median 16.2 [Interquartile range, IQR: 15.9, 17.3] AU) (Fig. 1a). The levels and the variation increased to a median 540 (IQR: 215, 2088) AU on Day 7, and the median fold change was 30.6 (IQR: 12.8, 90.7; p < 0.0001) (Fig. 2). Nineteen (90%) of the 21 included volunteers were considered to be responders.

We excluded the sublingual saliva specimens from one volunteer from analyses because the day-10 anti-YghJ IgA levels consistently increased (> 25%) when treated with nYghJ during GSP analyses described below. We suspect that in this volunteer the addition of nYghJ somehow freed IgA from mucoid aggregates that disturbed the measurement and could not be removed by size exclusion chromatography. The corresponding median anti-YghJ IgA levels in saliva were 697 (IQR: 373, 1377) nAUs before infection and 1432 (IQR: 502, 2594) nAUs on Day 10 (Fig. 1b), and the median fold change was 1.86 (IQR: 1.13, 3.71; p = 0.006) (Fig. 2) for the 20 volunteers included in these analyses. Only 10 (50%) of the volunteers were considered to be responders, including 5 who contributed whole saliva and 5 who contributed sublingual saliva. There was no difference in median total IgA concentration between whole and sublingual saliva. (Detailed results are given in supplementary data2 and 3.). Having experienced diarrhea during the infection did not seem to be strongly associated with increased anti-YghJ IgA levels in ALS, saliva, intestinal lavage or serum. For ALS, the median fold changes were 65.5 (IQR: 34.7, 151) and 27.4 (IQR: 11.5, 50.8) for those who did and did not develop diarrhea, respectively, (p = 0.13) while the corresponding fold changes for saliva were 1.69 (IQR: 1.28, 3.06) and 2.03 (IQR: 1.03, 3.08) (p = 0.97).

Magnitude of fold changes of anti-YghJ IgA isolated from ALS and saliva were evaluated with previously published measurements for intestinal lavage and serum [28].

﻿To evaluate whether the fold change in IgA antibody levels correlated between different specimen types, we calculated Pearson correlation coefficients on the log10-transformed fold change estimates in anti-YghJ IgA among the responders, which included 19 volunteers for ALS, 10 for saliva, 14 for intestinal lavage, while 20 were included for serum analyses.

**Fig. 3:**
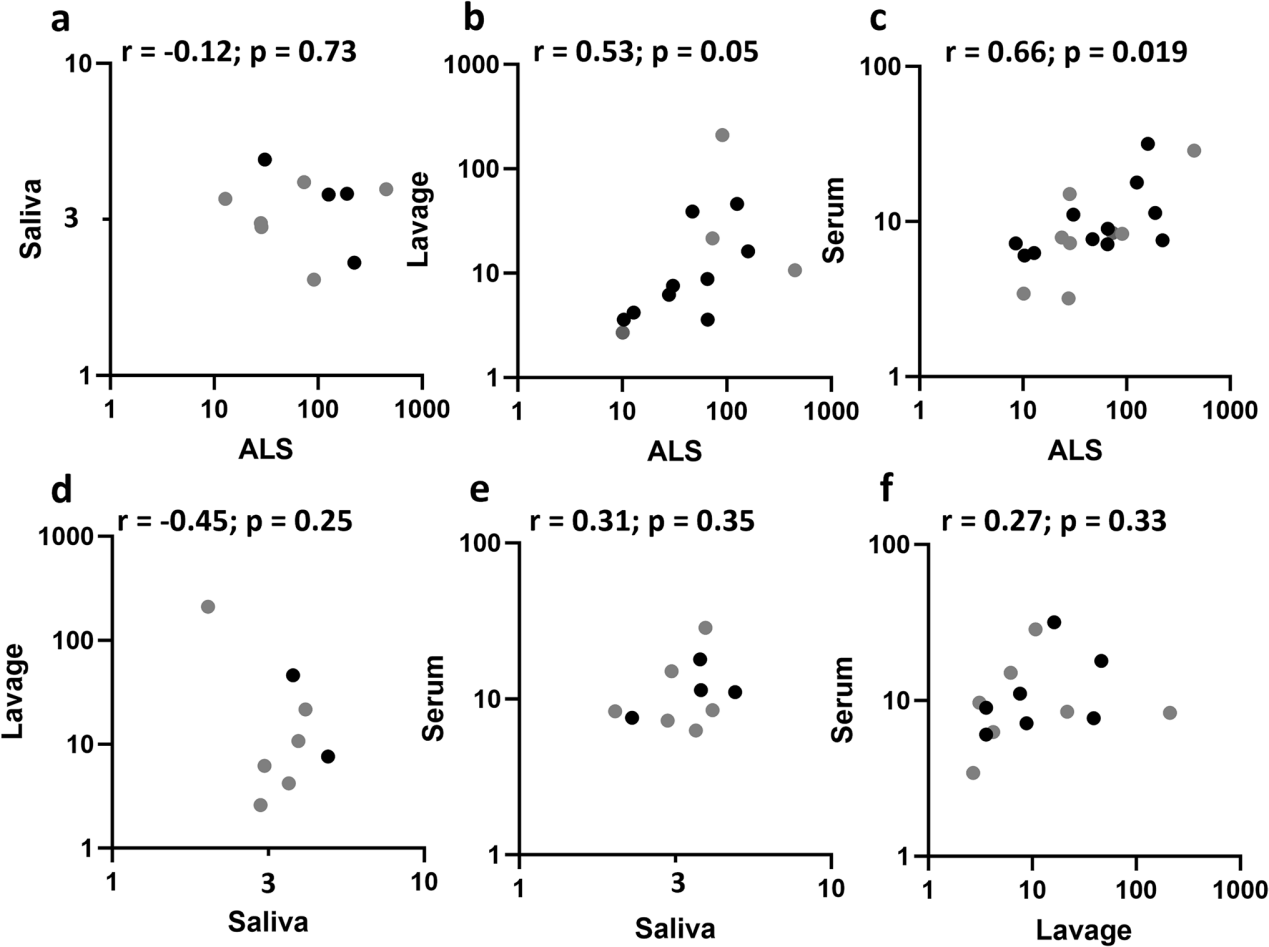
Correlation of log fold change in anti-YghJ IgA levels between different specimen types among responders. The r denotes the Pearson’s correlation coefficient and p the corresponding p-values. Black and grey dots represent volunteers who did and did not develop diarrhea, respectively

The fold change in ALS anti-YghJ IgA levels seemed to be strongly correlated with those in serum (Fig. 3c), while its correlations with saliva and intestinal lavage (Fig. 3a and b), respectively, were less clear. The fold change in saliva did not seem to be correlated with those in lavage or with serum (Fig. 3d and e), although the number of comparisons in the former analyses were relatively small (8 volunteers). No clear correlations existed between intestinal lavage and serum specimens (Fig. 3f). These correlations did not seem to change substantially when tested on post-infection IgA levels rather than the fold changes estimates (supplementary data 1). Finally, there was no group level difference in fold change correlations between volunteers who developed diarrhea and those who did not (data not shown).

### Anti-YghJ IgA target specificity

**Fig. 4:**
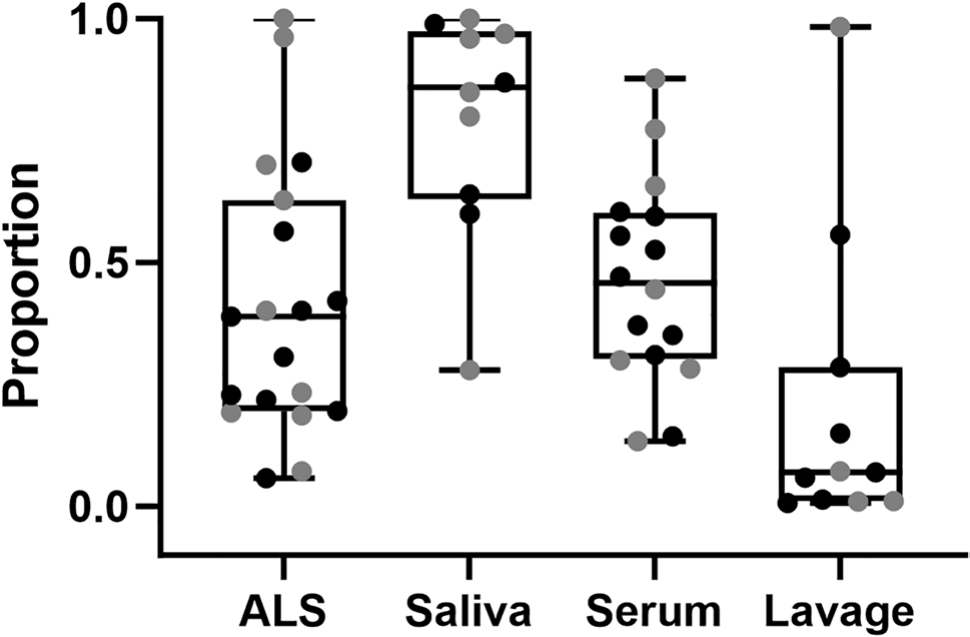
Boxplots of the glycosylation-specific proportions (GSP) of anti-YghJ IgA from different specimen types. Line in the boxes represents median values and boxes the values between 25 and 75th percentiles. The upper and lower whiskers limit 95% of measured values. Black and grey dots represent volunteers who did and did not develop diarrhea, respectively

We estimated the proportions of anti-YghJ IgA that targeted glycosylated YghJ epitopes (GSP) in the 19 ALS and 10 saliva specimens that had been collected from the responders on Day 7 (for ALS) and Day 10 (for saliva). The median GSP was 0.39 (IQR: 0.20, 0.59; range: 0.05, 1.04) for ALS and 0.86 (IQR: 0.68, 0.96; range: 0.28, 1.01) for saliva (Fig. 4). There was no clear difference in the median GSP between saliva specimens from volunteers who contributed whole saliva and from those who contributed sub-lingual saliva (p = 0.54). The corresponding, already published [28], estimates for the 11 intestinal lavage and 16 serum samples were 0.07 (IQR: 0.01, 0.22; range: 0.01, 0.98) and 0.45 (IQR: 0.30, 0.59; range: 0.13, 0.88), respectively (Fig. 4). The median GSP for saliva was substantially higher than that for intestinal lavage (p = 0.0008), while the median GSP for ALS did not seem to differ clearly from that of serum (p = 0.42).

Consistent to what was earlier found for intestinal lavage and serum [28], there was no significant difference in GSP between volunteers who did and did not develop diarrhea both for ALS and saliva.

**Fig. 5:**
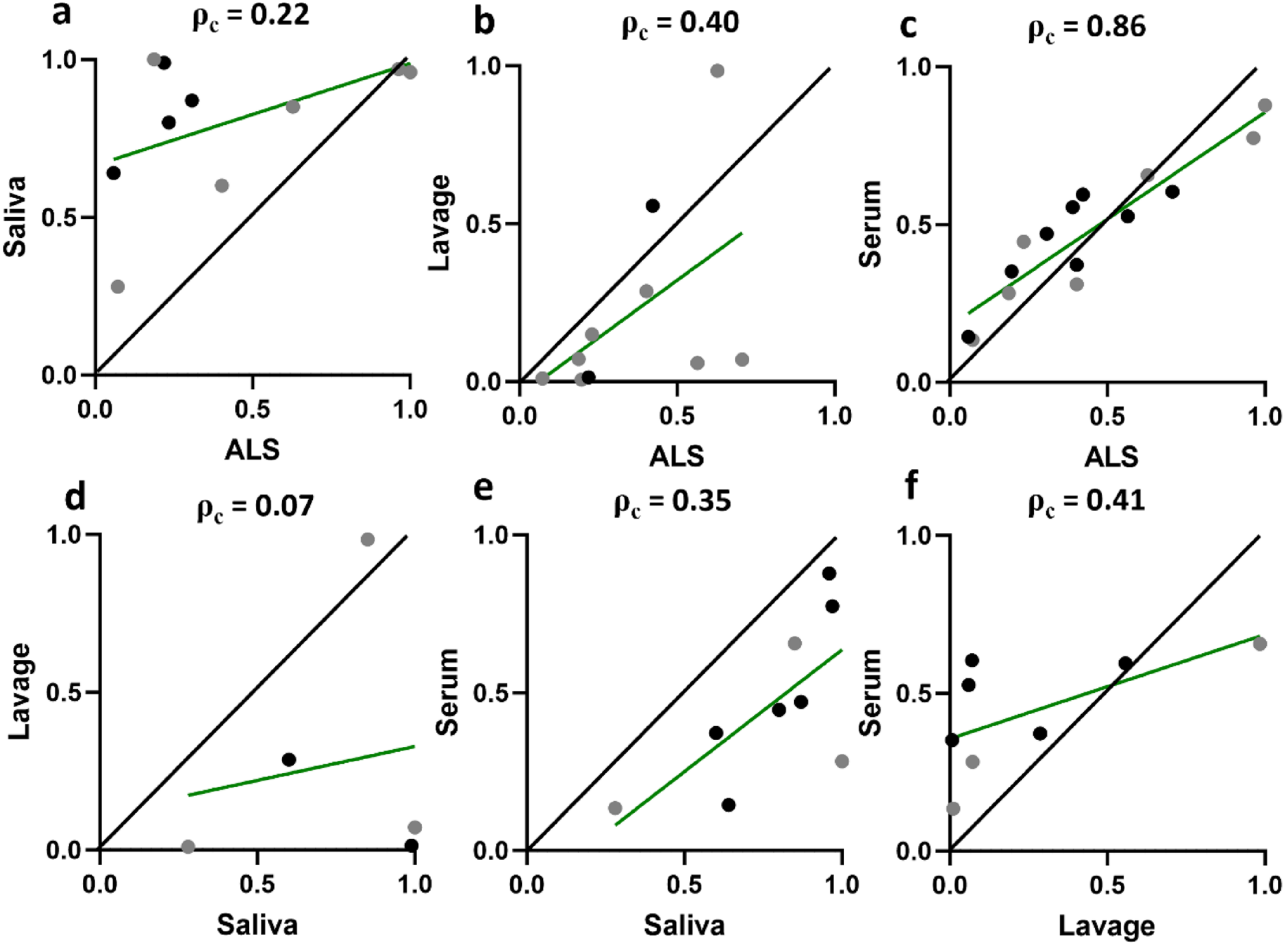
Concordance correlation analyses of the glycosylation-specific proportion (GSP) of anti-YghJ IgA in different specimen types. The axes indicate the GSP for each given specimen type. The p_c_ values are the estimated Lin’s concordance correlation coefficients. The black, solid 45-degree lines indicate what a perfect agreement would look like. The green lines indicate the best fit linear regression line of the data point. Black and grey dots represent volunteers who did and did not develop diarrhea, respectively

We evaluated the extent of agreement between GSP of anti-YghJ IgA isolated from ALS and saliva with the previously published estimates for intestinal lavage and serum. The clearest agreement in GSP was between ALS and serum (p_c_ = 0.86 [95% Confidence Interval, CI: 0.71, 0.93]) (Fig. 5c). There were little agreements between ALS and saliva (p_c_ = 0.22 [95% CI: – 0.10, 0.51]) (Fig. 5a), ALS and intestinal lavage (p_c_ = 0.40 [95% CI: – 0.13, 0.75]) (Fig. 5b), saliva and serum (p_c_ = 0.35 [95% CI: – 0.02, 0.64]) (Fig. 5e), and serum and intestinal lavage (p_c_ = 0.41 [95% CI: – 0.06, 0.73]) (Fig. 5f), while the agreement was poor between saliva and intestinal lavage (p_c_ = 0.07 [95% CI: – 0.42, 0.53]) (Fig. 5d). Consistently, we did not find a difference between diarrhea and non-diarrhea cases with regards to correlation between different matrices (data not shown).

## Discussion

Results from a recent experimental ETEC infection study suggested that only a small proportion of intestinally secreted anti-YghJ IgA targeted glycosylated epitopes [28]. In the present study we found that anti-YghJ IgA from saliva and ALS had a clearly higher proportion of anti-YghJ IgA targeting the glycosylated epitopes than the intestinal IgA, and that the glycosylation specificity of anti-YghJ IgA in ALS seemed to correlate with those in serum.

It is believed that gut IgA originates from the same recirculating plasmablasts that also circulate in blood [18], but our findings suggest that the recirculating plasmablasts found in blood are not necessarily representative of the plasma cells that give rise to the intestinal anti-YghJ IgA antibody response. Similarly, the lack of correlation both in fold-change and in anti-YghJ specificity between IgA isolated from saliva and intestinal lavage suggests that the antibody-secreting cells at the two mucosal sites have been subject to selection or modulation.

Antigen activated B cells migrating to the effector sites may be selectively extravasated on the basis of complementary adhesion molecules and chemokine-chemokine receptor pairs [34]. Furthermore, terminal differentiation of B cells to plasma cells in mucosal tissues is dependent on topical exposure of antigens [34]. Final localization of plasma cells apparently takes place within the segments of the gut where they were initially activated [35, 36]. As a result, there could be site-specific accumulation of specific IgA plasma cells and variation in antibody specificities at different secretory sites [37, 38].

The reason that the target specificity in saliva specimens differs from that of intestinal lavage could partly be attributed to the suggestion that nasopharynx-associated lymphoid tissue, such as adenoids and palatine tonsils, are more important than gut-associated lymphoid tissue as inductive sites for B cells migrating to salivary glands [14, 39]. While B cell extravasation into the gut lamina propria is dependent on the lymphocyte integrin α4β7 [40], this integrin is not important for homing to salivary glands [37]. Nevertheless, the reason salivary YghJ-specific IgA antibodies seem to target glycosylated epitopes warrants additional investigation.

Our findings also suggest that it would be simplistic to assume that vaccine candidate antigens that are natively glycosylated will produce an equivalent antibody response in their recombinant non-glycosylated forms. Additional studies are needed to expand our understanding of host–pathogen glycan interactions and resulting adaptive immune responses pertinent to vaccine candidate antigens.

A study of human salivary responses found that the magnitude of the salivary secretory IgA response against whole ETEC bacteria correlated with the intestinal immune response after experimental infection [14], and since saliva collection is simple and minimally invasive, using saliva IgA as a proxy for gut IgA seems an attractive option [13]. However, these responses against homologous whole cell bacteria are likely mainly targeting the variable LPS based O-antigen, while in our study the specific IgA responses against the conserved protein antigen YghJ were much less pronounced in saliva than in lavage specimens. Thus, even if ETEC infection may lead to a strong response in both saliva and lavage for abundant and strong antigens, the antibody response against a single virulence protein antigen differed in these two matrixes, as did its specificity for glycosylated epitopes.

A low glycosylation-specific proportion of anti-YghJ IgA in lavage could also be ascribed to possible depletion of IgA antibodies targeting glycosylated epitopes due to cross-reactions with other glycosylated microbial target proteins in the gut [41]. Also, lavage specimens reflect the combined secretory IgA response of both the small and large intestine, where small intestinal IgA may, perhaps, be masked by the plasma cell repertoire of the large intestine [6, 37].

### Limitations

Immune responses in saliva and lavage can be harder to standardize and interpret. Many of the same challenges are present when working with intestinal lavage specimens and saliva specimens, including the need for filtration/purification procedures, and the need to normalize IgA levels before analyses due to variable IgA secretion and/or recovery [31, 42–44]. For example, in saliva specimens we developed a size-exclusion chromatography method using Sephadex G-25 and used MagPlex beads, allowing better washing steps, to counter high background signals. However, we do not expect that bead choice should affect IgA targeting glycosylated YghJ epitopes differently from IgA targeting non-glycosylated YghJ epitopes in the same specimen, thereby biasing our GSP estimates. Intra- and inter assay variation may have affected our results.

For our saliva GSP analyses we combined results from whole saliva and sublingual saliva specimens since both specimen types contained IgA from the submandibular and sublingual glands. Although we did not see a statistically significant difference in GSP between the two specimen types, the specimen counts were too small to conclude unequivocally that they are the same.

Our focus has been on YghJ antigens [29, 45], in particular to what extent IgA from different types of specimens target glycosylated epitopes of YghJ, and it may not be a general rule for other natively glycosylated virulence factors, e.g. EtpA [46], AIDA-I [47], and type 1 fimbriae [48]. Further research is needed to evaluate whether our findings are specific for glycosylated YghJ, or they can be generalized to other glycosylated *E. coli* virulence factors, or whether the variation in target specificities can also be found for other post-translational protein modifications.

The strength of using ALS specimens is its ability to control for the confounding effect of accumulative antibodies in serum specimens that contain both recent and pre-existing soluble antibodies [15]. However, the lack of normalization against the quantity of plasmablasts in each ALS specimen may have lowered the precision of the ALS response magnitude.

Finally, for GSP correlation analyses between ALS and saliva or lavage, the low number of responders limited our ability to firmly assess the correlation between these specimens. However, this is also a general limitation of human challenge studies where, due to ethical and technical concerns, the number of volunteers is usually small [49].

### Conclusion

We found that experimental ETEC infection elicits a strong anti-YghJ IgA response in humans, but the magnitude and glycosylation-specific anti-YghJ IgA response differs substantially between recirculating plasmablasts and mucosal secretions. This finding suggests that the plasmablasts circulating in the blood are not uniformly distributed along the gastrointestinal tract. It remains to be identified when and how, during antibody maturation process, the target specificity of mucosally secreted IgA changes.

We also found that the intestinally secreted anti-YghJ IgA showed different target specificities to those of anti-YghJ IgA secreted in saliva. Our findings suggest that neither IgA from saliva, ALS, or serum may accurately reflect the composition of intestinally secreted IgA, and that it may therefore be difficult to assume that these types of specimens are suitable proxies for studying intestinally secreted antibodies. Further studies are needed for a better insight into the mechanisms involved in intra-mucosal compartmentalization and contrasting predilections of antibodies towards glycosylated epitopes through in-depth characterization of antibodies or plasma cell clones from oral and intestinal biopsies.

## Supplementary Information

Below is the link to the electronic supplementary material.

Supplementary file1 (DOCX 208 KB)

## Data Availability

No datasets were generated or analysed during the current study.
