# Supplementary material for: Proportions of IgA antibodies targeting glycosylated epitopes of secreted Escherichia coli mucinase YghJ in initial plasmablast response differ from salivary and intestinally secreted IgA
Source: Med Microbiol Immunol. 2024 Dec 14;214(1):2. doi: 10.1007/s00430-024-00812-0 (PMC11646272; doi:10.1007/s00430-024-00812-0)
Supplement: Supplementary file 1 — Supplementary file1 (DOCX 208 KB) [file 430_2024_812_MOESM1_ESM.docx]

**Proportions of IgA antibodies targeting glycosylated epitopes of secreted *Escherichia coli* mucinase YghJ in initial plasmablast response differ from salivary and intestinally secreted IgA**

Saman Riaz ^1^, Hans Steinsland ^2,3^, Ann Z. Andersen^4^, Anders Boysen^4^, Kurt Hanevik ^1,5^*

^1^ Department of Clinical Science, University of Bergen, Bergen, Norway

^2^ Centre for Intervention Science in Maternal and Child Health (CISMAC), Centre for International Health, Department of Global Public Health and Primary Care, University of Bergen, Bergen, Norway

^3^ Department of Biomedicine, University of Bergen, Bergen, Norway

^4^ GlyProVac ApS, Rørhatten 4, Odense, Denmark

^5^ Norwegian National Advisory Unit on Tropical Infectious Diseases, Department of Medicine, Haukeland University Hospital, Bergen, Norway

***** Correspondence: kurt.hanevik@uib.no

**Supplementary Data**

**1. Supplementary Fig. 1 Correlation of post-infection anti-YghJ IgA levels between different specimens’ types among responders**


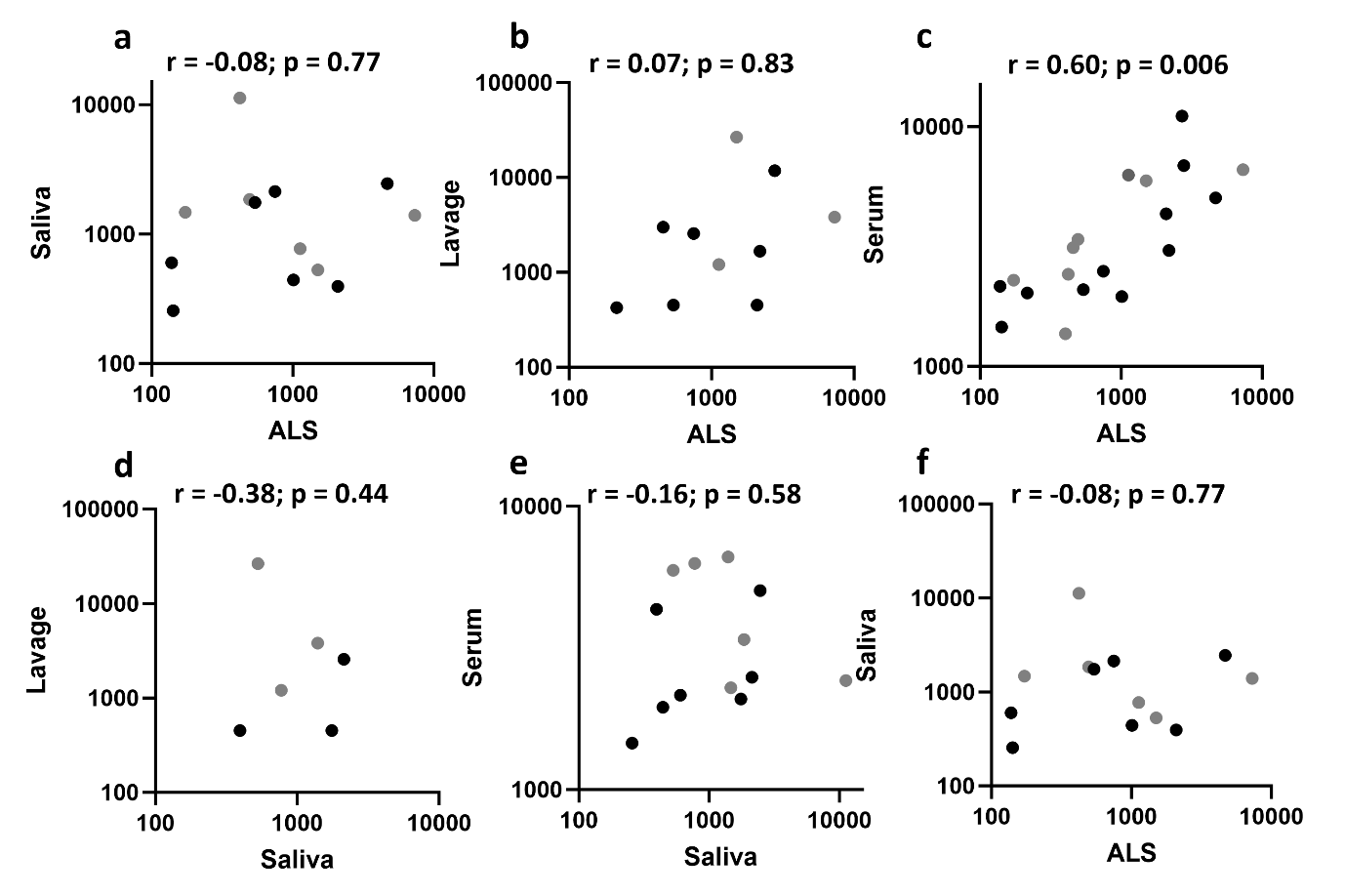


The r denotes the Pearson's correlation coefficient and p the corresponding p-values. Black and grey dots represent volunteers who did and did not develop diarrhea, respectively. ALS and serum values are presented in AU, and saliva and lavage in nAU.

**2. Total saliva IgA concentrations – comparisons between whole and sublingual saliva**

We collected whole saliva from the first 9 volunteers and sublingual saliva from the last 11 both immediately before and 10 days after dose ingestion. For the whole saliva specimens collected from the 9 volunteers, the median total IgA concentration on Day 0 (median 0.30 [Interquartile range (IQR): 0.26, 0.51; range: 0.23, 1.07] mg/mL) did not differ substantially from those collected on Day 10 (median 0.47 [IQR: 0.25, 1.15; range: 0.23, 7.13] mg/mL) (p = 0.12). Correspondingly, there was also no clear difference in the median IgA concentration in sublingual saliva specimens collected from the remaining 11 volunteers (0.27 [IQR: 0.17, 0.33; range: 0.05, 1.44] mg/mL on Day 0 and 0.27 [IQR: 0.22, 0.35; range: 0.12, 1.22] mg/mL) on Day 10 (p = 0.45). Overall, the combined total IgA in whole saliva at Day 0 and Day 10 (median 0.29 (IQR: 0.25, 0.75) mg/mL) did not seem to differ substantially from the combined total IgA in sublingual saliva at Day 0 and Day 10 (median 0.27 (IQR: 0.20, 0.35) mg/mL; p = 0.12).

**3. Supplementary Table. 1 Total saliva IgA (mg/mL) data used to normalize anti-YghJ saliva IgA levels, anti-YghJ IgA levels and GSP after normalization.**

| **ID** | **Saliva total IgA levels (mg/ml)** | | **Anti-YghJ IgA levels (AU)** | | **GSP after normalization** |
| --- | --- | --- | --- | --- | --- |
|  | **Day 0** | **Day 10** | **Day 0** | **Day 10** | **Day 10** |
| EV01 (WS) | 0.24 | 0.25 | 143 | 74 | NA |
| EV02 (WS) | 0.28 | 0.47 | 448 | 2341 | 0.957 |
| EV03 (WS) | 0.23 | 1.16 | 407 | 603 | NA |
| EV04 (WS) | 0.49 | 0.14 | 91 | 106 | 0.848 |
| EV05 (WS) | 0.3 | 0.84 | 2415 | 9404 | NA |
| EV06 (WS) | 0.52 | 0.23 | 421 | 741 | 0.277 |
| EV07 (WS) | 0.26 | 0.27 | 100 | 373 | 0.966 |
| EV08 (WS) | 1.07 | 2.56 | 1393 | 1535 | NA |
| EV09 (WS) | 0.72 | 7.13 | 151 | 3021 | 1.011 |
| EV19 (SLS) | 0.17 | 0.22 | 217 | 471 | NA |
| EV21 (SLS) | 0.17 | 0.21 | 78 | 112 | NA |
| EV22 (SLS) | 1.44 | 1.22 | 407 | 541 | NA |
| EV23 (SLS) | 0.46 | 0.37 | 363 | 1122 | 0.599 |
| EV24 (SLS) | 0.3 | 0.84 | 323 | 2067 | 0.642 |
| EV25 (SLS) | 0.28 | 0.27 | 101 | 475 | 0.988 |
| EV26 (SLS) | NA | NA | NA | NA | NA |
| EV27 (SLS) | 0.27 | 0.26 | 133 | 384 | 0.796 |
| EV28 (SLS) | 0.13 | 0.28 | 59 | 71 | NA |
| EV29 (SLS) | 0.25 | 0.31 | 84 | 121 | NA |
| EV30 (SLS) | 0.38 | 0.35 | 940 | 3349 | 0.872 |
| EV31 (SLS) | 0.05 | 0.12 | 90 | 223 | NA |
| Median | 0.28 | 0.28 | 546 | 1372 | 0.78 |

***WS = whole saliva; SLS = Sub-lingual saliva; GSP = Glycosylation-specific proportion; NA = Not applicable**
